# A supervised term ranking model for diversity enhanced biomedical information retrieval

**DOI:** 10.1186/s12859-019-3080-2

**Published:** 2019-12-02

**Authors:** Bo Xu, Hongfei Lin, Liang Yang, Kan Xu, Yijia Zhang, Dongyu Zhang, Zhihao Yang, Jian Wang, Yuan Lin, Fuliang Yin

**Affiliations:** 1 Faculty of Electronic Information and Electrical Engineering, Dalian University of Technology, Linggong Road, Dalian, People’s Republic of China; 2 WISE Lab, School of Public Administration and Law, Dalian University of Technology, Linggong Road, Dalian, People’s Republic of China; 3 State Key Laboratory of Cognitive Intelligence,iFLYTEK, Hefei, People’s Republic of China

**Keywords:** Biomedical information retrieval, Supervised query expansion, Term ranking model, Diversity-oriented retrieval, Machine learning, Learning to rank

## Abstract

**Background:**

The number of biomedical research articles have increased exponentially with the advancement of biomedicine in recent years. These articles have thus brought a great difficulty in obtaining the needed information of researchers. Information retrieval technologies seek to tackle the problem. However, information needs cannot be completely satisfied by directly introducing the existing information retrieval techniques. Therefore, biomedical information retrieval not only focuses on the relevance of search results, but also aims to promote the completeness of the results, which is referred as the diversity-oriented retrieval.

**Results:**

We address the diversity-oriented biomedical retrieval task using a supervised term ranking model. The model is learned through a supervised query expansion process for term refinement. Based on the model, the most relevant and diversified terms are selected to enrich the original query. The expanded query is then fed into a second retrieval to improve the relevance and diversity of search results. To this end, we propose three diversity-oriented optimization strategies in our model, including the diversified term labeling strategy, the biomedical resource-based term features and a diversity-oriented group sampling learning method. Experimental results on TREC Genomics collections demonstrate the effectiveness of the proposed model in improving the relevance and the diversity of search results.

**Conclusions:**

The proposed three strategies jointly contribute to the improvement of biomedical retrieval performance. Our model yields more relevant and diversified results than the state-of-the-art baseline models. Moreover, our method provides a general framework for improving biomedical retrieval performance, and can be used as the basis for future work.

## Background

Biomedical research has developed rapidly in recent years, which leads to a dramatic increase in the number of biomedical research articles. The huge amount of articles make it more and more difficult for researchers to obtain the needed information. Information retrieval (IR) technologies have therefore been introduced to fulfill the information needs. Given a certain query, biomedical retrieval systems aim to provide users with a ranking list of relevant articles. However, the information needs can hardly be fully satisfied due to the ambiguity and uncertainty of queries. This is because the submitted user queries always contain biomedical terminologies in different forms. Namely, a certain terminology may be associated with different synonyms, acronyms and polysemies, which increases the difficulty of term matching in biomedical IR systems. As a result, existing IR methods cannot easily achieve desired performance when directly being applied to solve the problem. Retrieval performance can be enhanced by accurately matching the query terms and potentially relevant terms from articles.

To tackle the problem, biomedical IR systems aim to retrieve the most relevant articles to the given queries, and meanwhile address the diversity of search results for completely meeting the information needs [1, 2]. The relevance of articles measures the similarity between a given query and the retrieved results. The diversity of the search results is characterized by the covered query-related aspects in the outputted ranking list. Each aspect for a given query can satisfy the information need from a certain perspective. All the aspects of the query capture the complete information needs. Therefore, the ratio of covered aspects is used to measure the diversity of search results in biomedical information retrieval. The goal of biomedical IR is thus transformed to retrieve relevant documents covering as many aspects of the query as possible. An aspect of a query can be described with certain biomedical terms, and all the aspects of a query constitute a complete interpretation of the information needs. For example, the biomedical query ’How does P53 affect apoptosis?’ involves query-related aspects, such as ’apoptosis regulatory proteins’, ’tumor suppressor protein P53’ and ’gene expression’. To completely answer the biomedical query, biomedical IR aims to retrieve the most relevant and diversified documents covering these aspects.

Existing studies have been carried out to improve the relevance and the diversity of biomedical search results [3–9]. However, few studies have addressed the diversity-oriented biomedical IR using query expansion methods. Query expansion is an effective technique used in IR tasks, which enriches user queries by adding useful terms for interpreting the information needs. General query expansion methods are divided into two categories: unsupervised query expansion (UQE) and supervised query expansion (SQE). UQE methods have been studied for years and have been successfully applied to solve biomedical IR tasks, which measure the importance of expansion terms based on a certain predefined scoring function. The effectiveness of UQE methods varies a lot when using different scoring functions. In fact, different scoring functions measure the usefulness of candidate expansion terms from different perspectives, thus achieving diverse performance.

To completely capture the useful expansion terms, SQE methods have been proposed recently, and effectively improved the quality of the expanded queries [10–13]. SQE methods integrate different UQE methods as term features for learning a supervised model. The model is trained based on a classifier or a ranker for further term refinement. Recent studies have indicated that SQE methods are advantageous over UQE methods in terms of two respects. On one hand, SQE methods count the term importance from different perspectives, which can be considered as the combination of various UQE methods for term refinement. On the other hand, SQE methods can better deal with the retrieval tasks under certain constraints. Since biomedical retrieval focuses on the diversity constraints of search results, we believe that SQE methods can be well adapted to enhance the performance of biomedical IR using effective optimization strategies.

In this paper, we propose a novel supervised term ranking model based on supervised query expansion for diversity-oriented biomedical information retrieval. Our model seeks to obtain the most relevant and diversified search results in biomedical IR. Three optimization strategies are integrated in the proposed model. A biomedical term labeling strategy is designed to capture the relevance and the diversity degrees of the candidate expansion terms.Both the context-based and resource-based term features are extracted to reflect the usefulness of different terms. A group sampling method is proposed to capture the diversity during the model training process. We modify the loss function of ranking support vector machines using the group sampling method with a diversity-oriented weighting function to improve the quality of selected expansion terms. We evaluate the proposed model on the collections from the TREC Genomics tracks. Experimental results show that our model is effective in improving the performance of biomedical IR.

We summarize the main contributions of this work as follows.

(1) We introduce supervised query expansion for diversity-oriented biomedical information retrieval, and propose a novel method to improve retrieval performance.

(2) We propose to annotate the usefulness of expansion terms by considering the relevance and the diversity simultaneously, and extract abundant features for term representations.

(3) We integrate the group sampling and diversity-oriented weighting function into the loss function of ranking support vector machines to improve the quality of expansion terms.

## Related work

Query expansion aims to enrich user queries to comprehensively fulfill user information needs in information retrieval (IR), which have been widely used in different IR tasks. Expansion term selection is one of the challenging research topics in query expansion. The quality of selected terms largely affects the accuracy of query expansion. How to select effective terms has attracted much attention in related studies. For example, Lee et al. [14] captured the underlying term associations using abundant term features based on linguistics and statistics. Cao et al. [10] classified the candidate expansion terms based on support vector machines to distinguish good terms from bad terms for expansion. Furthermore, learning-to-rank methods have been investigated to refine expansion terms [13, 15]. These supervised machine learning methods for query expansion have been proved to be effective in improving retrieval performance by considering multiple term features, called supervised query expansion. In this study, we mainly focus on term refinement based on modified supervised query expansion.

Existing studies in biomedical information retrieval have integrated query expansion to improve retrieval performance. For example, Srinivasan [16] evaluated the effectiveness of query expansion on MEDLINE collections using the SMART retrieval system. Xu et al. [17] compared query expansion techniques involving local analysis, global analysis and ontology for biomedical literature retrieval. Matos et al. [18] developed a document retrieval and prioritization tool using concept-oriented query expansion to obtain documents with respect to related concepts. Rivas et al. [19] investigated query-specific terms, corpus-specific terms and language-specific terms for biomedical query expansion. These studies demonstrated that query expansion can enhance biomedical IR by considering domain-specific characteristics.

Furthermore, other studies have focused on latent concept expansion in medical and clinical IR tasks, which have also been addressed in general IR tasks. These studies indicated that latent expansion concepts can positively affect biomedical retrieval performance. For example, Bendersky et al. [20] assigned weights on candidate concepts using a weighted dependence model for improving retrieval effectiveness. Zhu et al. [21] identified patient cohorts using mixtures of relevance models to weight query expansion terms for clinical search. TREC 2011 and 2012 medical records tracks have also addressed concept-based retrieval for vertical domain retrieval [22]. In addition, related studies have employed biomedical semantic resources, particularly the MeSH thesaurus, in query expansion. For example, Oh et al. [23] incorporated the structure of external collections to optimize pseudo relevance feedback. Mao et al. [24] integrated a MeSH-enhanced concept layer into a language modeling framework to capture concept associations. Jalali et al. [25] matched concept pairs between queries and documents using a semantic query expansion method. These studies motivate us to optimize query expansion in consideration of domain knowledge.

Inspired by the related work, we propose a supervised query expansion method for diversity-oriented biomedical information retrieval. The proposed method modify our learn-to-rank based query expansion framework [26] from two respects. One is assigning diversity-oriented term labels and extract different types of resource-based term features. The other is developing a novel learning method based on group sampling and diversity weighting function.

## Methods

### General framework

In this section, we introduce more details about our supervised query expansion framework for diversity-oriented biomedical information retrieval. The pipeline of our framework is illustrated in Table 1. Our framework includes two stages: the training stage and the testing stage. In the training stage, we adopt pseudo relevance feedback (PRF) to obtain a large set of candidate expansion terms for each training query. We then represent each candidate expansion term as a feature vector with a ground truth label. In these vectors of terms, we extract both context-based features and resource-based features, and label terms in consideration of their relevance and diversity. We adopt supervised ranking methods to refine the terms by modifying loss function for diversity. In the testing stage, we apply the learned model to select the expansion terms for query expansion.

**Table 1:** Supervised query expansion for biomedical information retrieval

Algorithm 1 supervised query expansion pipeline
Training the SQE model *M*
1: For each training query *q*, select *k* candidate terms via PRF
2: Label each term based on the diversity-oriented strategy
3: Represent each term as a feature vector using different term features
4: Train term ranking model *M* using the modified loss function
Testing the model *M* in query expansion retrieval
1: For each testing query *q*, select *k* candidate terms via PRF
2: Represent each term as a feature vector using the term features
3: Apply *M* to obtain the top *m* terms for query expansion

We introduce the details of our proposed method in the following sections, including candidate term extraction, term labeling strategy, term features and learning model construction.

### Candidate term extraction

For each query, a set of candidate expansion terms are extracted for further refinement. The candidate expansion terms should be highly correlated with the given query in terms of both relevance and diversity. We adopt a modified pseudo relevance feedback method [26] to extract the terms. The method has been proved to be effective in biomedical information retrieval, which considers term distribution in feedback documents and term distribution in Medical Subject Headings (MeSH) to extract useful expansion terms for further refinement.

### Term labeling strategy

The term labeling strategy is designed to assign ground truth labels on the candidate expansion terms. The labels are treated as the learning targets, and used to compute the ranking loss during model training. A well-defined labeling strategy is crucial for learning a well-performed term ranking model. Existing labeling strategies of supervised query expansion are mostly based on term relevance to a given query. The term relevance can be measured based on its influence on retrieval performance [10]. Specifically, we first conduct an initial retrieval with the original query *q*, and record the retrieval performance as *Eval*(*q*) by any evaluation metric *Eval*, such as mean average precision. We then conduct another retrieval with both *q* and a given candidate term *t*, and record its performance as *Eval*(*q*,*t*). The relevance label for *t* is determined by comparing *Eval*(*q*) with *Eval*(*t*,*q*). This method can be formalized as follows.

1 $$ \begin{aligned} label(t)=\left\{\begin{aligned} &0\ \ \ \ Eval(t,q)\leq Eval(q) \\ & 1\ \ \ \ Eval(t,q)> Eval(q) \end{aligned} \right. \end{aligned}  $$

Since the diversity is an important factor for biomedical IR, relevance-based labeling may be insufficient for measuring the usefulness of terms. Therefore, we consider the diversity degrees of terms in generating ground truth labels. Query diversity can be reflected by query-related aspects. Aspects of a given query are explicitly described using domain-specific terms in biomedical IR. Therefore, we can measure the diversity of terms based on their occurrences in query-related aspects.

An intuitive way is to generate the diversity-oriented term labels by considering whether a certain term is contained in any query-related aspect. If the term is contained in any query-related aspect, we believe the term is useful and assign the label 1 to the term. Otherwise, we assign the label 0 to the term. Although this labeling strategy seems simple and feasible, it may ignore some potentially useful information of terms: a term contained in several aspects tends to be more diversified than other terms contained in only one aspect. Besides, the influence of terms on retrieval performance is still an important factor in term labeling. Based on the above consideration, we present a new labeling strategy that integrates the diversity and the relevance of terms, which is formulated in Table 2.

**Table 2:** Diversity-oriented term labeling strategy

Relevance/Diversity	*div*(*t*)=0	*div*(*t*)=1	*div*(*t*)>1
*Eval*(*q*,*t*)>*Eval*(*q*)	1	2	3
*Eval*(*q*,*t*)≤*Eval*(*q*)	0	1	2

In the table, *div*(*t*) is the number of the query-related aspects containing the term *t*. The aspects of queries have been manually annotated in advance on benchmark collections, such as TREC Genomics Tracks, for the diversity-oriented biomedical retrieval task. More aspects containing *t* indicate that the term can cover more aspects of the query to diversify the search results. Term labels are divided into four types: definitely useful (label 3), partly useful (label 2), probably useful (label 1) and not useful (label 0). The strategy with multiple labels can more accurately measure the ranking loss than that with binary labels, thus producing more effective term ranking models.

### Term features

Terms are represented as feature vectors for learning supervised term ranking models. Each term feature corresponds to one term statistic, reflecting the term usefulness for the given query. We extract two types of term features in our method: the context-based features and the resource-based features. We introduce the definitions of these two feature sets below.

#### Context-based features

Context-based features consider the distribution of terms within the retrieval collection. Since textual statistics are always used to measure the term distribution, we adopt different textual statistics as term features. Two types of features are extracted in our framework: features based on term frequency and inverse document frequency (*tfidf*) and features based on co-occurrences (*cooc*). These features have been proved effective in our previous work [13].

For the *tfidf* based features, term frequency (*tf*), inverse document frequency (*idf*) and their combination are treated as different features. Term frequency measures the number of occurrences of certain terms in a document. Inverse document frequency measures the number of documents with a certain term. These two textual statistics can be combined as *tfidf* to jointly measure term importance within the entire retrieval collection. We extract these *tfidf* based features both within the entire collection and the top-ranked feedback documents from initial retrieval as different term features.

For the *cooc* based features, term co-occurrences with query terms are considered. Intuitively, if a certain term co-occurs frequently with query terms, the term is more likely to be treated as an expansion term for query enrichment. Therefore, we extract the *cooc* features based on the number of co-occurrences. We not only limit the scope of co-occurrences in the document level, but also use sliding windows to extract fine-grained term features.

We extract these context-based features to measure term importance and relevance based on term distribution in the retrieval collection. Furthermore, we extract some resource-based features to capture the domain-specific characteristics of biomedical candidate expansion terms.

#### Resource-based features

There exist a large amount of semantic resources for biomedical text mining. These resources contain abundant semantic and syntactic information of biomedical terminologies. The resource-based information can be used for modeling the relationship among domain-specific terms. Therefore, we propose to extract domain-specific term features based on biomedical resources. Two widely used resources, Medical Subject Headings (MeSH) and MetaMap, are investigated in our work.

MeSH has been widely used to index and catalog biomedical articles on biomedical search engines, such as PubMed. The terminologies in MeSH are organized in a tree-based hierarchical manner. The term distribution in MeSH can reflect term importance in biomedicine. To capture the term information from MeSH, we define two indicators in analogy to the term frequency and inverse document frequency used in IR tasks. We name these two indicators as MeSH-based term frequency (*tf*_*MeSH*_) and MeSH-based concept frequency (*idf*_*MeSH*_), respectively. MeSH-based term frequency accumulates the occurrences of certain terms in MeSH. If a certain term occurs frequently in MeSH, it may be important in the biomedical domain, and contains much domain-specific information. We define the MeSH-based term frequency as follows.

2 $$ \begin{aligned} {tf}_{MeSH}(t_{j})=\frac{log(freq(t_{j},MeSH)+1.0)}{log|T|} \end{aligned}  $$

where |*T*| represents the number of terms in MeSH. *freq*(*t*_*j*_,*MeSH*) counts the number of occurrences of *t*_*j*_ in MeSH.

MeSH-based concept frequency accumulates the number of unique biomedical concepts containing a certain term in MeSH. If more concepts contains a certain term, the term is more likely to reflect domain-specific characteristics. Specifically, this indicator is defined as follows.

3 $$ \begin{aligned} {idf}_{MeSH}(t_{j})=\frac{M-m(t_{j})+1.0}{m(t_{j})+1.0} \end{aligned}  $$

where *M* is the number of concepts, and *m*(*t*_*j*_) is the number of unique concepts containing *t*_*j*_ in MeSH. *idf*_*MeSH*_(*t*_*j*_) measures the importance of *t*_*j*_ in MeSH.

Furthermore, inspired by the statistic *tfidf* used in IR, we combine *tf*_*MeSH*_(*t*_*j*_) and *idf*_*MeSH*_(*t*_*j*_) as a new term feature. The feature is defined as follows.

4 $$ \begin{aligned} {tfidf}_{MeSH}(t_{j})={idf}_{MeSH}(t_{j})log({tf}_{MeSH}(t_{j})+1.0) \end{aligned}  $$

Besides, we adopt MetaMap to extract more domain-specific term features. MetaMap is a powerful natural language processing tool in biomedicine. This tool has been widely applied in various biomedical text mining tasks [27]. MetaMap is designed by the National Library of Medicine (NLM) to detect domain-specific concepts from biomedical texts. The concepts are from the Unified Medical Language System (UMLS) metathesaurus. The detected concepts can reflect the domain characteristics of the original texts. We therefore extract term features based on the detected concepts from queries.

Specifically, we first expand the original query with a certain candidate expansion term as an expanded query. we then map the expanded query to a concept query using MetaMAP. The concept query contains the canonical forms of Concept Unique Identifiers (CUIs). Intuitively, if the concept query involves more biomedical concepts, the concept query is likely to convey more domain-specific information. The candidate expansion term may be more effective for query expansion. Hence, we define the number of detected concepts as a term feature. This feature is formalized as follows.

5 $$ \begin{aligned} concept(t)=count(t,Q_{expand}(t)) \end{aligned}  $$

where *Q*_*expand*_(*t*) is the expanded query with the candidate expansion term *t*. *count*(*t*,*Q*_*e*_*xpand*(*t*)) accumulates the number of occurrences of *t* in the concept representations of the expanded query. Since MetaMap returns several candidates for an expanded query, the number of returned candidates may also capture the term importance in biomedicine. We define two term features based on this idea.

6 $$ \begin{aligned} conceptnum(t)={count}_{CUI}(t,Q_{expand}(t)) \end{aligned}  $$

7 $$ \begin{aligned} candidate(t)=\frac{\sum\nolimits_{q \in Q_{expand}(t)}|R(c)|}{count_{CUI}(t,Q_{expand}(t))} \end{aligned}  $$

where *conceptnum*(*t*) measures the total number of concepts in the concept query at the query level. |*R*(*c*)| is the number of returned candidates for the concept *c* with respect to *Q*_*expand*_. We normalize the feature values by the number of concepts contained in the concept query to make the feature values comparable to each other.

All the candidate expansion terms are represented as feature vectors based on the context-based and resource-based features. The feature vectors are treated as the inputs for model training. The intermediate models are optimized based on pre-defined ranking loss functions towards the ground truth term labels.

### Group enhanced loss function for term ranking

In this section, we introduce the ranking loss function in our method. Loss function measures the difference between the intermediate predictions and their corresponding targets for model selection at the training time, which can be iteratively reduced until the optimal model is yielded. A well-performed loss function for biomedical term selection should count the difference between predicted term labels and the ground truth labels in consideration of both relevance and diversity degrees of terms. To this end, we introduce a group sampling method based on the group enhanced ranking algorithm [28]. The group enhanced ranking algorithm is an effective learning to rank method based on the divide-and-conquer strategy. Learning to rank methods have been widely used in the field of information retrieval to improve the ranking performance, which construct ranking models using supervised machine learning methods.

To adapt group enhanced ranking to biomedical term refinement, we divide the candidate expansion term sets for each query into smaller groups. Each term group contains one term with higher label and several terms with lower labels. We accumulate the losses produced by all the groups to achieve the total loss of intermediate model, and optimize the model by iteratively reducing the total loss. The loss is reduced by gradient descent, particularly on wrongly ranked groups. Therefore, we believe that the group sampling method can yield the final model that focuses on highly useful terms by ranking effective terms at the top of term ranking list, thus improving the quality of the expanded query. The loss function based on group sampling can be formalized as follows.

8 $$ \begin{aligned} loss(k)=\sum\limits_{i=1}^{m}\sum\limits_{j=1}^{n} L\left(f\left(t_{i}^{j},\omega\right),y_{i}\right) \end{aligned}  $$

where *m* is the number of queries and *n* is the number of groups for the *i*^*th*^ query. *f* is the predictive function and *y* is the target label. The function *L* characterizes the similarity between the predicted label $f(t_{i}^{j},\omega)$ and the target label *y*_*i*_. Different versions of *L* have been used in different learning to rank methods, such as the exponential function and the logarithmic likelihood function. Based on this function, we accumulate the ranking loss of every group of term samples. Since diversity is a key factor to choose the biomedical expansion terms, we further incorporate a diversity-oriented weights on groups to address the diversity of different groups. We formalize the method as follows.

9 $$ \begin{aligned} loss(k)=\sum\limits_{i=1}^{m}\sum\limits_{j=1}^{n} \left(\gamma\left(t_{i}^{j}\right)\right)L\left(f\left(t_{i}^{j},\omega\right),y_{i}\right) \end{aligned}  $$

where *γ* is the diversity-oriented weighting function. Different types of *γ* functions can be adopted in our framework with the underlying idea that more diversified terms should be chosen as expansion terms. We provide one type of *γ* function as follows.

10 $$ \begin{aligned} \gamma(t_{i}^{j})=\frac{1}{Z}\times\frac{NumAsp(t_{i}^{j})+1.0}{TotalCount(t_{i}^{j})+1.0} \\s.t.\ \ 0<\gamma<1 \end{aligned}  $$

where *NumAsp*(*t*) represents the number of aspects containing the term *t*. *TotalCount*(*t*) represents the number of occurrences of term *t* in all the aspects of the given query. *Z* is a normalization factor to limit the weights within the range of 0 to 1. Based on the above defined loss function, the constructed term ranking model pays more attention on relevant and diversified terms for in-depth query understanding and complements.

### Group enhanced ranking support vector machines for term ranking

Our diversity-oriented query expansion framework is flexible and can be implemented using different supervised learning methods. In this study, we adopt the ranking support vector machines (RankSVM) [29] to examine the performance of our model using the loss function in Eq. (9).

RankSVM is a variant of the support vector machine algorithm, which is used to solve certain ranking problems via learning to rank. The original purpose of the algorithm was to improve the performance of an internet search engine. The goal of RankSVM is to learn a linear model to minimize the number of pairs of terms in wrong preference orders. Formally, the objective function of RankSVM is defined as follows.

11 $$ \begin{aligned} &min\frac{1}{2}\omega^{T}\omega+C\sum\limits_{i=1}^{m}\sum\limits_{j=1}^{n}\sum\limits_{u,v,y_{u,v}^{i}}\xi_{u,v}^{i,j}\\ &s.t.\ \ \omega^{T}\left(t_{u}^{i,j}-t_{v}^{i,j}\right)\geq 1-\xi_{u,v}^{i,j},t_{u}^{i,j}\succ t_{v}^{i,j},\xi_{u,v}^{i,j}\geq 0 \end{aligned}  $$

where $t_{u}^{i,j}\succ t_{v}^{i,j}$ implies that the term *u* should be ranked ahead of the term *v* in the *j*^*th*^ group for the *i*^*th*^ query. *C* is the trade-off coefficient between the ranking loss and the model complexity.

To apply the group enhanced ranking to RankSVM, we incorporate the diversity-oriented weighting function into the objective function. The modified objective function seeks to compute the ranking loss in consideration of the diversity and relevance of terms. The final form of the objective function is defined as follows.

12 $$ \begin{aligned} &min\frac{1}{2}\omega^{T}\omega+C\sum\limits_{i=1}^{m}\sum\limits_{j=1}^{n}\sum\limits_{u,v,y_{u,v}^{i}}\xi_{u,v}^{i,j}\\ &s.t.\ \ \gamma\left(t_{u}^{i,j}\right)\omega^{T} t_{u}^{i,j}\geq \gamma\left(t_{v}^{i,j}\right)\omega^{T} t_{v}^{i,j} +1\\ &\ \ \ \ \ \ \ \ \ \ -\xi_{u,v}^{i,j},t_{u}^{i,j}\succ t_{v}^{i,j},\xi_{u,v}^{i,j}\geq 0 \end{aligned}  $$

where *γ* is the diversity-oriented weighting function based on group sampling. This modified objective function is used to compute the ranking loss as the function *L* in Eqs. (8) and (9) does for model construction. We believe that the learned ranking model based on the modified objective function can select more relevant and diversified terms for biomedical query expansion. The well-constructed expanded queries will contribute to enhancing the retrieval performance.

## Results

In this section, we evaluate the proposed model with sufficient experiments. We first introduce the experimental settings, and then evaluate the performance in terms of labeling strategy, term feature and loss functions, respectively. We finally report the overall retrieval performance of our framework and provide analysis and discussions on the results.

### Experimental settings

We evaluated the proposed framework on the retrieval collections from 2006 and 2007 TREC Genomics tracks [1, 2]. The collections are public available and contain 162,259 articles from 49 biomedical journals. The objective for these tracks is to design effective information retrieval systems, which can search for relevant articles and passages given biomedical queries. There are totally 26 queries for 2006 track and 36 queries for 2007 track.

We adopted four evaluation measures, Document MAP, Passage MAP, Passage2 MAP and Aspect MAP. These measures were designed for the tracks as variations of Mean Average Precision (MAP). MAP is a classic evaluation measure used in general IR tasks. The former three measures were designed to evaluate retrieval performance in terms of document-level relevance and passage-level relevance. Aspect MAP aimed to evaluate the retrieval performance in terms of the diversity of the search results.

We implemented the proposed method within the Indri search engine framework [30]. Indri, as a general IR framework, has been widely used in different IR tasks. We indexed articles from the experimental collections with stemmed words and stopword removed in advance. We tuned the parameters of our method for 2006 collection with 2007 queries, and tuned the parameters for 2007 collection with 2006 queries. The selected parameters are reported in Table 3, which have been used in our previous work [26].

**Table 3:** Parameter settings in our method

Para.	#Feedback docs.	#Expa. terms	#Cand. terms
2006	60	30	150
2007	10	30	150

To build ranking models based on RankSVM, we performed five-fold cross validations. The reported performance is the average performance on all the five folds. We divided the training set, testing set and validation set by query numbers at the ratio of 3:1:1. The training set was used for model training, the testing set was used for prediction, and the validation set was used for ranking parameter selection. The division follows the standard partition for the learning-to-rank datasets in LETOR [31]. Next, we evaluate the labeling strategy, term feature and loss functions, respectively, and report the overall retrieval performance and discussions.

### Performance of labeling strategies

We conducted the first experiment to examine the effectiveness of the proposed labeling strategy compared to two baseline strategies: One is based on the relevance of terms using Eq. (1), denoted as BinaryRel, and the other is based on whether a term is contained in query-related aspects, denoted as BinaryDiv. We report the retrieval performance in Table 4.

**Table 4:** Effectiveness of different labeling strategies

	Document	Passage	Passage2	Aspect
2006 queries				
BinaryRel	0.3129	0.0221	0.0271	0.2579
BinaryDiv	0.3095	0.0237	0.0337	0.2635
The Proposed	0.3282	0.0249	0.0345	0.2828
2007 queries				
BinaryRel	0.3091	0.0796	0.1093	0.2552
BinaryDiv	0.3252	0.0845	0.1153	0.2721
The Proposed	0.3337	0.0847	0.1155	0.2723

The table shows that the binary relevance strategy achieved relatively good performance in terms of the relevance-based measure Document MAP, while the binary diversity strategy achieved good performance in terms of the diversity-based measure Aspect MAP. This indicates that term labels can guide the model learning process and improve retrieval performance from different respects. Furthermore, the proposed strategy outperformed the other two strategies in terms of both Document MAP and Aspect MAP. This observance indicates that the proposed labeling strategy considering both the relevance and diversity of terms are effective for improving the retrieval performance. One explanation for this finding is that more relevant and diversified terms can be achieved for expansion when the model is optimized towards relevance and diversity of term labels.

### Performance of term features

We conducted the second experiment to examine the effectiveness of different sets of term features, including the context-based set (Context), the resource-based set (Resource) and the combination set with all the features (All). We report the experimental results in Table 5 on the two collections.

**Table 5:** Effectiveness of different feature sets

	Document	Passage	Passage2	Aspect
2006 queries				
Context	0.3168	0.0317	0.0288	0.2613
Resource	0.3130	0.0238	0.0335	0.2713
All	0.3282	0.0249	0.0345	0.2828
2007 queries				
Context	0.3037	0.0786	0.1076	0.2428
Resource	0.3126	0.0804	0.1096	0.2605
All	0.3337	0.0847	0.1155	0.2713

From the table, we observe that for 2006 queries, the context-based features outperformed the resource-based features in terms of Document MAP, but less performed in terms of Aspect MAP. The model using all the features achieved the best performance by all the evaluation measures. For 2007 query, the model with the resource-based features outperformed that based on the context-based features, and the model using all the features was the most effective in improving the performance. These results implies that both the context-based features and the resource-based features can contribute to the overall performance of the term ranking model. These two feature sets are complementary to each other and jointly improve the performance by combining them together.

### Performance of loss functions

We conducted the third experiment to examine the effectiveness of the proposed loss function in comparison with other loss functions, and report retrieval performance in Table 6. In the table, original ranking loss represents that the model was trained based on the original form of the loss function of RankSVM, group sampling loss represents that the model was trained based on the loss function of RankSVM with group sampling, and weighted group loss represents the proposed form of loss function in Eq. (12).

**Table 6:** Effectiveness of different ranking loss functions

	Document	Passage	Passage2	Aspect
2006 queries				
Original loss	0.3065	0.0235	0.0335	0.2632
Group loss	0.3088	0.0235	0.0335	0.2641
Proposed loss	0.3282	0.0249	0.0345	0.2828
2007 queries				
Original loss	0.3226	0.0844	0.1160	0.2667
Group loss	0.3307	0.0841	0.1153	0.2684
Proposed loss	0.3337	0.0847	0.1155	0.2713

The table shows that group sampling indeed enhanced the performance of the original RankSVM, and the diversity-oriented term weighting further achieved the best performance. The experimental results indicates that group samples of terms help distinguish the useful terms and useless terms, thus enhancing retrieval performance. The diversity-oriented term weighting further guides the model training for more diversified terms, and improve the diversity of retrieval results in terms of aspect MAP to a large extent.

### Overall retrieval performance

In this section, we report the overall retrieval performance of our model based on the proposed labeling strategy, all the defined features and the weighted loss function with group sampling. We compared our model with state-of-the-art baseline models.

For the models compared, the query-likelihood language model [32] is a classic retrieval model in IR. The language model is also used as the basic retrieval model in our experiments. Relevance model [33] and term dependency model [15] are two unsupervised query expansion models widely used in different tasks. Support Vector Machine (SVM), RankSVM and ListNet [34] are three learning to rank methods belonging to the pointwise approach, the pairwise approach and the listwise approach, respectively. The SVM-based SQE method has been proved effective in [10]. We report the retrieval performance of all the models in Table 7 on the two collections. Two-tailed paired Student t-tests (*p*<0.05) were used to examine whether the improvements are significant relative to the baseline models. In the table, an asterisk (∗) indicates significant improvements over the RankSVM-based model and a dagger (*†*) indicates significant improvements over the ListNet-based model.

**Table 7:** Overall retrieval performance of different models for 2006 queries

	Document	Passage	Passage2	Aspect
2006 queries				
Language model [32]	0.3178	0.0205	0.0239	0.1983
Relevance model [33]	0.3194	0.0207	0.0240	0.2023
Term dependency [15]	0.3198	0.0208	0.0254	0.1785
SVM-based SQE [10]	0.3050	0.0237	0.0292	0.2447
ListNet [34]	0.3216	0.0234	0.0290	0.2256
RankSVM [29]	0.3065	0.0235	0.0335	0.2632
Our model	0.3282* *†*	0.0249* *†*	0.0345* *†*	0.2828* *†*
2007 queries				
Language model [32]	0.2587	0.0646	0.0876	0.2000
Relevance model [33]	0.2678	0.0720	0.0963	0.2302
Term dependency [15]	0.2804	0.0683	0.0939	0.1974
SVM-based SQE [10]	0.2833	0.0729	0.0999	0.2298
ListNet [34]	0.2819	0.0739	0.1012	0.2255
RankSVM [29]	0.3226	0.0844	0.1160	0.2467
Our model	0.3337* *†*	0.0847* *†*	0.1155 *†*	0.2713* *†*

In the table, an asterisk (*) indicates significant improvements over the RankSVM-based model and a dagger (*†*) indicates significant improvements over the ListNet-based model

The table shows that compared to classic retrieval models, unsupervised query expansion methods enhanced the retrieval performance of biomedical retrieval task. Furthermore, the supervised query expansion method improved the performance on both query sets. Our method significantly outperformed other baseline models in terms of most evaluation measures, which demonstrates the effectiveness of the proposed model. One possible explanation for this finding is that all the modifications of the proposed method based on RankSVM contribute to constructing an effective diversity-oriented term ranking model for choosing high-quality expansion terms to expand the original query, and interpret the query for better fulfilling the information needs in biomedical information retrieval.

To further evaluate our retrieval performance, we compared our results with the median results, the mean results and the best results reported in the 2006 and 2007 tracks of TREC Genomics. The results of the comparisons are presented in Table 8.

**Table 8:** comparison with the best and mean results in the Genomics tracks

	Document	Passage	Passage2	Aspect
2006 queries				
Median MAP	0.3083	0.0316	0.0345	0.1581
Mean MAP	0.2887	0.0347	0.0392	0.1643
Best MAP	0.5439	0.1012	0.1486	0.4411
Our model	0.3282	0.0249	0.0345	0.2828
2007 queries				
Median MAP	0.1897	0.0565	0.0377	0.1311
Mean MAP	0.1862	0.0560	0.0398	0.1326
Best MAP	0.3286	0.0976	0.1148	0.2631
Our model	0.3337	0.0847	0.1155	0.2713

The table shows that the proposed model largely improved the mean and the median results of 2006 official submissions in terms of Document MAP and Aspect MAP, and outperformed the best result of 2007 official submissions in terms of most evaluation measures. The results imply that our method enhanced the relevance and diversity of biomedical retrieval results. We also observe that our method achieved better results in terms of Document MAP and Aspect MAP, but did not perform as well by Passage MAP and Passage2 MAP. This is because these two passage-level measures evaluate the retrieval results at character-level precision, which require extra processing by splitting the retrieved documents into relevant pieces. Our method does not seek to optimize this step, and therefore yields lower performance in terms of these measures. We will optimize our method to enhance these measures in our future work for fine-grained retrieval results.

### Discussion

In this work, we introduce supervised query expansion for term refinement in diversity-oriented biomedical information retrieval. Our model annotates the usefulness of expansion terms by simultaneously considering the relevance and the diversity of terms. The context-based and resource-based features are extracted for comprehensive term representation. In model training, we incorporate the group sampling and diversity-oriented weighting function into the loss function of ranking support vector machines to improve the quality of expansion terms. Overall, we attribute the improvement of the proposed method in biomedical information retrieval in three respects: the term-labeling strategy, the term features and the ranking models. For the term-labeling strategy, we propose to consider the query-related aspects to generate the ground truth labels of candidate terms, which yields more accurate labels during model training. For the term features, we extract both the context-based and the resource-based features, which depict the usefulness of terms more completely from different perspectives and complement each other in constructing the term-ranking models. For the ranking models, we introduce the group sampling and diversity-oriented weights to learn more effective term ranking models. These three aspects jointly contribute to the improvement in retrieval performance, and the proposed framework can also be further optimized in these aspects to enhance biomedical retrieval performance.

## Conclusions

In this study, we propose a novel supervised term ranking model to address the diversity-oriented biomedical information retrieval task. Our model is constructed based on the supervised query expansion process. The learned model integrates three novel optimization strategies to select the most relevant and diversified terms for query enrichment. We first propose a diversity-oriented term labeling strategy by considering the diversity degrees of terms. We then represent the candidate expansion terms using both the context-based and resource-based features. To enhance the learned models, we incorporate the group sampling method with a diversity-oriented weighting function into the ranking loss function of RankSVM. Experimental results on TREC collections demonstrate the effectiveness of the proposed model. Our model outperforms the baseline models, and effectively improves the performance of biomedical information retrieval in terms of relevance and diversity. Our model provides a general framework for improving biomedical retrieval performance. Our future work will seek to extract more powerful term features based on other useful biomedical resources, and investigate other effective supervised learning methods for further optimizing the proposed framework.

## Data Availability

Not applicable.
